# Molecular docking analysis of KRAS inhibitors for cancer management

**DOI:** 10.6026/97320630019411

**Published:** 2023-04-30

**Authors:** Israa J. Hakeem, Fatmah Hazza Alsharif, Majidah Aljadani, Ibrahim Fahad Alabbas, Mohammed Saud Faqihi, Ahmed Hamdan Aloufi, Wael Abdullah Almutairi, Asif Hussain Akber, Qamre Alam

**Affiliations:** 1 Department of Biochemistry, College of Science, University of Jeddah, Jeddah, Saudi Arabia; 2 Department of Medical Surgical Nursing Oncology and Palliative Care Nursing, Faculty of Nursing, King Abdulaziz University, Jeddah, Saudi Arabia; 3 Department of Chemistry, College of Sciences and Arts, King Abdulaziz University, Rabigh, Saudi Arabia; 4 Central Military Laboratory and Blood Bank Department - Virology Division, Prince Sultan Military Medical City, Riyadh 12233, Saudi Arabia; 5 Central Military Laboratory and Blood Bank Department - Microbiology Division, Prince Sultan Military Medical City, Riyadh 12233, Saudi Arabia; 6 Department of Pathology and Laboratory Medicine, Imam Abdulrahman bin Faisal Hospital Ministry of National Guard Health Affairs, P.O. Box 34232 Dhahran, Saudi Arabia; 7 Department of Respiratory Services, Ministry of National Guard Hospital and Health Affairs (MNGHA) P.O. box 22490, kingdom of Saudi Arabia; 8 Central Military Laboratory and Blood Bank Department - Virology Division, Prince Sultan Military Medical City, Riyadh 12233, Saudi Arabia; 9 Molecular Genomics and Precision Medicine Department, ExpressMed laboratories, Block, 359, Zinj, Kingdom of Bahrain

**Keywords:** Cancer, KRAS, natural compounds, inhibitors, virtual screening

## Abstract

The majority of human tumors are characterized by abnormal signaling caused by oncogenic RAS proteins. KRAS is a member of the RAS family and is currently one of the most thoroughly researched targets for cancer treatment due to its prevalence in a variety of deadly malignancies. Targeting the KRAS protein, which plays a crucial role in regulating cell growth, differentiation, and apoptosis, shows great potential as a strategy for fighting cancer. Herein, in silico screening of 530 natural compounds against KRAS protein was performed. The top-scoring hits, namely ZINC32502206, ZINC98363763, ZINC85645815, and ZINC98364259 displayed a robust affinity towards KRAS as evidenced by their respective binding affinity values of -10.50, -10.01, -9.80, and -9.70 kcal/mol, respectively which were notably higher than that of the control compound AMG 510 (-9.10 kcal/mol). Through virtual screening and visual inspection, it was observed that these hits effectively interacted with the essential residues located within the active site of KRAS. Based on the findings of this study, it can be inferred that these compounds may have the potential to be employed in the treatment of cancer by targeting KRAS.

## Background:

Cancer was responsible for nearly 10 million deaths worldwide in 2020, and it remains one of the leading causes of death worldwide. The World Health Organization (WHO) estimated in 2019 that cancer ranks among the top two causes of death before the age of 70 in 112 out of 183 countries, while in 23 other countries, it is classified as the third or fourth leading cause of death [1]. Targeted therapies have significantly transformed the treatment and outcomes of cancer, which is presently the primary cause of mortality worldwide [2]. The primary reason for cancer is assumed to be mutations in the cellular genome. One family of genes, RAS, including NRAS, HRAS, and KRAS, is commonly associated with mutations in various types of human tumors, including those found in the pancreas, colon, lung, thyroid, and myeloid leukemia [3]. These genes play a role in a range of signaling pathways, such as cell growth and differentiation, by encoding a group of GTPases [4]. Annually, approximately one million people die as a result of KRAS mutations alone worldwide [5].

The normal development of cells, including proliferation and differentiation, relies significantly on the vital role played by KRAS [6]. KRAS, being a small GTPase, undergoes a cyclic transition between an inactive state, which is GDP-bound, and an active state, which is GTP-bound. This transition is precisely regulated by two distinct types of proteins, namely GTPase-activating proteins and Guanine nucleotide exchange factors, which are responsible for inactivating and activating KRAS, respectively [7,8]. Mutant KRAS, on the other hand, hinders the activity of its GAPs, which results in KRAS being locked in an active state [9]. This abnormal behaviour of mutant KRAS leads to the promotion of its interaction with a range of effector proteins, which, in turn, triggers downstream signalling events that culminate in the formation of tumors [10,11]. There is an urgent need to identify effective inhibitors that can target and block oncogenic KRAS in cancers.

Computer-aided drug design plays a crucial role in predicting potential targets and compounds during the process of discovering new compounds, as well as in evaluating biological competencies and optimizing drug activity [12]. This study aimed to screen natural compounds (NPs) against the KRAS protein in order to find a KRAS inhibitor to fight cancer.

##  Methodology:

## Target protein retrieval and preparation:

Human KRAS crystal structure (PDB ID: 6OIM) was obtained from the Protein Data Bank. The protein's native structure was bound to the Sotorasib (AMG 510) ligand, which was resolved at a low resolution of 1.65 Å [13]. The co-crystal ligand and the water molecules were eliminated from the protein, which was subsequently subjected to minimization and prepared using Discover Studio before being utilized in the virtual screening (VS) process.

## Preparation of compounds for screening:

For this study, a collection of 530 NPs was assembled from the ZINC database. These NPs were obtained in their original '.sdf' format and were subjected to ligand preparation via energy minimization using the mmff94 force field. The resulting ligand structures were then saved in 'pdbqt' format to facilitate subsequent screening procedures.

## Virtual screening:

In drug discovery and preclinical research, VS has emerged as a potentially effective tool to reduce the time, cost, and resource requirements significantly [14]. The primary objective of screening is to search chemical compounds databases to identify new hits with the most promising biological activity [15]. We used the PyRx 0.8 tool [16] to screen the prepared NPs library against the KRAS active site. Following that, we conducted a thorough interaction analysis and visual inspection to identify the most stable complex based on lower binding energy (BE) values.

## Physicochemical and ADMET Properties prediction:

Small molecule drug discovery and therapeutics depend heavily on ADMET properties, which include absorption, distribution, metabolism, excretion, and toxicity. Insufficient ADMET properties have been implicated in the failure of numerous clinical trials. However, it is preferable to profile ADMET early in the drug discovery process because the experimental evaluation of ADMET properties is both pricey and data-restricted. To achieve this, this study employed a web server known as ADMETboost to predict the physicochemical and ADMET properties of four selected compounds [17].

## Results and Discussion:

**Figure 1:**
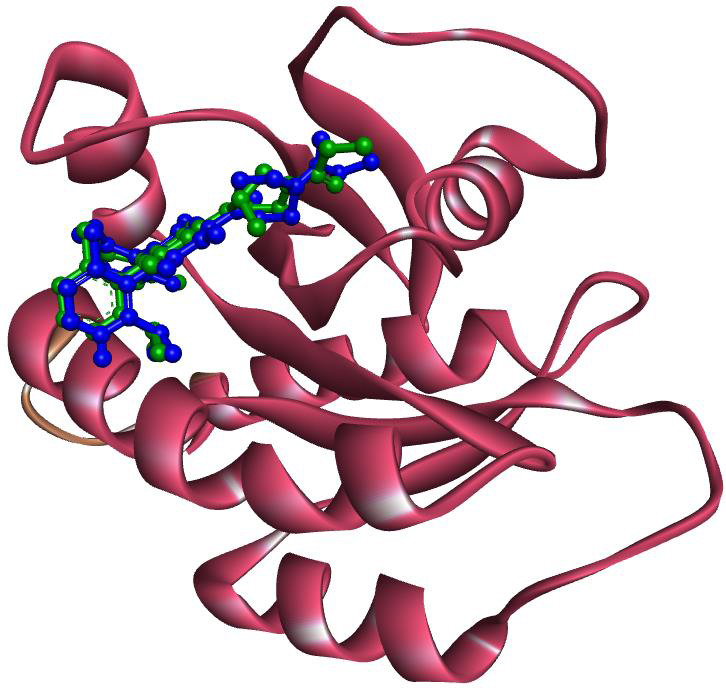
Superimposed binding mode of X-ray-bound AMG 510 (green) and re-docked AMG 510 (blue) with KRAS protein.

The KRAS protein crystal structure used in this screening was obtained in its biological complex with the authorized inhibitor AMG 510 (PDB ID: 6OIM). The validation of the docking protocol involved the removal of the crystal ligand (AMG 510) from the KRAS protein complex, followed by re-docking the ligand with the protein. The pose of the re-docked ligand was then computed, and it was observed that similar to co-crystal ligand binding orientation, AMG 510 also binds in the same binding pocket of the KRAS protein (Figure 1). This finding confirms the validity of the docking protocol in predicting the binding orientation of ligands in the binding pocket of the KRAS protein.

**Figure 2:**
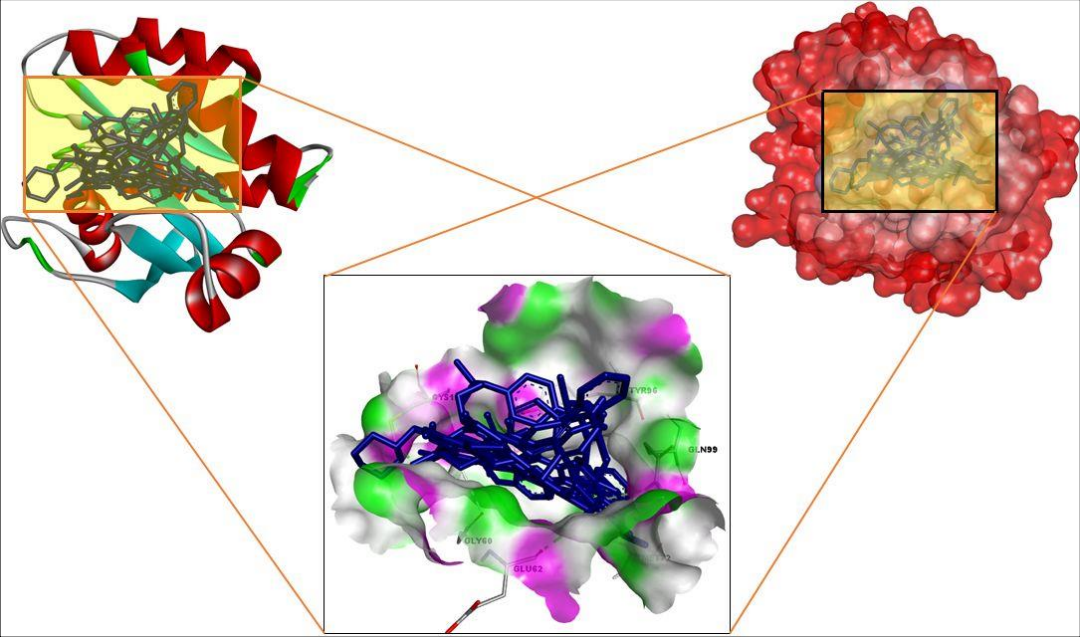
3D visualization of screened compounds in the active pocket of KRAS.

Here in this study, we performed a computational screening of 530 natural compounds targeting the active site residues of KRAS to identify novel natural inhibitors. VS revealed several potential compounds with higher BEs than the positive control AMG 510. (Table 1, Table 2). We report in this study four compounds that demonstrated more effective binding by interacting with critical KRAS residues, based on a detailed analysis and visualization of the docked complexes' interactions (Figure 2).

To determine the physicochemical, ADME, and toxicity properties of the four chosen natural compounds, ADMETboost was employed. ADMETboost is a web server that utilizes an ensemble of features, including fingerprints and descriptors, alongside a tree-based machine learning model. This approach allowed for accurate prediction of the properties of the natural compounds. The predictions indicate that all of the identified hits possess a significant range of ADMET properties and have the potential to be developed into drug molecules.

**Figure 3:**
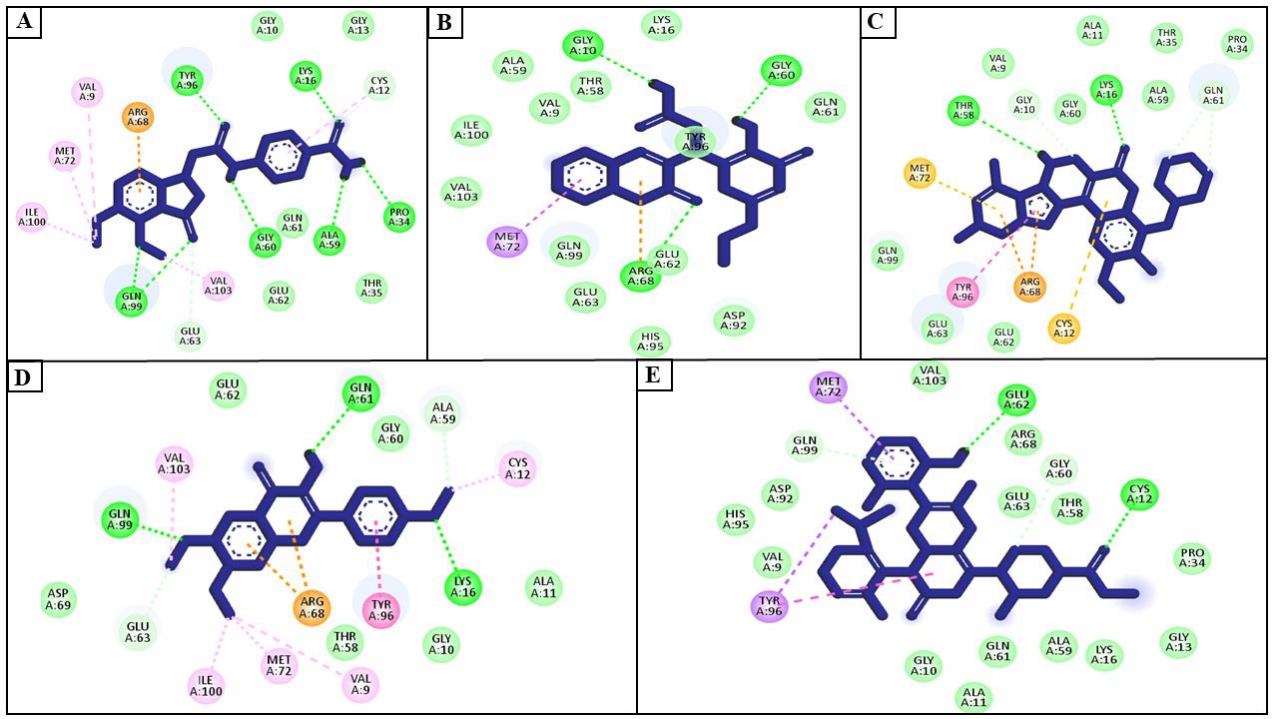
Interacting residues of KRAS protein with ZINC98364259 (A), ZINC98363763 (B), ZINC32502206 (C), ZINC85645815 (D), and AMG 510 (E)

The details interaction profile of the top 4 compounds with KRAS protein was analyzed. ZINC98364259 interacted with Arg68, Tyr96, Gly10, Lys16, Gly13, Cys12, Pro34, Ala59, Thr35, Gln61, Gly60, Glu62, Val103, Glu63, Gln99, Ile100, Met72, and Val9 residues of KRAS protein. Tyr96, Lys16, Pro34, Ala59, Gly60, and Gln99 residues were H-bonded with ZINC98364259 (Figure 3A). ZINC98363763 bind with Met72, Val103, Ile100, Val9, Ala59, Thr58, Gly10, Lys16, Gly60, Gln61, Tyr96, Glu62, Asp92, Arg68, His95, Glu63, and Gln99 residues of KRAS protein. The compound ZINC98363763 have H-bonds with Arg68, Gly10, and Gly60 residues of KRAS as shown in Figure 3B. ZINC32502206 interacted with Met72, Thr58, Val9, Gly10, Gly60, Lys16, Ala11, Thr35, Ala59, Gln61, Pro34, Cys12, Arg68, Glu62, Tyr96, Glu63, and Gln99 residues of KRAS protein. Thr58, and Lys16 residues were H-bonded with ZINC32502206 (Figure 3C). Further, ZINC85645815 interacted with Val103, Glu62, Gln61, Gly60, Ala59, Cys12, Ala11, Lys16, Gly10, Tyr96, Thr58, Val9, Arg68, Met72, Ile100, Glu63, Asp69, and Gln99 residues of KRAS protein. Gln99, Gln61, and Lys16 residues were H-bonded with ZINC85645815 (Figure 3D).

The KRAS G12C mutation has been identified as a promising drug target due to its capacity to engage in covalent binding with a cysteine residue (Cys12) and form hydrophobic interactions with other residues present in the cryptic allosteric pocket of the KRAS G12C protein. This binding results in the protein being locked in an inactive GDP-bound state, thus rendering it a potential target for therapeutic intervention [13, 18, 19]. Interestingly, similar to AMG 510, ZINC98364259, ZINC32502206, and ZINC85645815 compounds demonstrated interactive binding with Cys12, the reactive residue of the KRAS protein.

AMG 510 displayed anti-tumor effectiveness in the initial dose cohorts in clinical studies and represents a potentially transformational therapy for patients who lack viable therapies [13]. AMG 510 interacted with Met72, Val103, Glu62, Arg68, Gly60, Glu63, Thr58, Cys12, Pro34, Gly13, Lys16, Ala59, Gln61, Ala11, Gly10, Tyr96, Val9, His95, Asp92, and Gln99 residues of KRAS protein (Figure 3E). Interestingly, Met72, Glu62, Arg68, Gly60, Glu63, Lys16, Ala59, Gln61, Gly10, Tyr96, Val9, and Gln99 were the common binding residues of KRAS protein with top four compounds (ZINC98364259, ZINC98363763, ZINC32502206, and ZINC85645815) and the positive control AMG 510 (Figure 3A-3E).

The BE value indicates the degree of interaction between the compound and the protein complex, with a higher (negative) value indicating stronger binding to the target [20, 21]. In this regard, it is worth noting that the identified hits (ZINC98364259, ZINC98363763, ZINC32502206, and ZINC85645815) have higher binding affinity than AMG 510, implying that these compounds have a strong interaction with the KRAS protein.

The current cancer therapies and drugs have adverse side effects and are unsuitable for prolonged use. Therefore, the medical community strongly advocates for the use of alternative medicine, particularly natural compounds, as they offer effective treatment with minimal side effects. Plant-derived phytochemicals are interesting candidates for improving cancer treatment efficacy and reducing unwanted effects. Several of these natural compounds are biologically active substances found in nature with high anticancer potential [22]. A large body of research suggests that phytochemicals are important in the prevention and treatment of cancer and epidemiological studies suggest that increasing phytochemical intake may lessen the incidence of cancer. The mechanism of action has been elucidated through experimental research, which includes decreasing cancer cell proliferation, stimulating apoptosis and autophagy, and inhibiting angiogenesis and cancer cell metastasis [23]. The hits in this study are natural compounds that have been proposed to inhibit cancer progression through their interaction with the KRAS protein.

## Conclusion:

KRAS is a well-documented therapeutic target for cancer due to its widespread occurrence in a variety of life-threatening malignancies. The current study showed that ZINC98364259, ZINC98363763, ZINC32502206, and ZINC85645815 have high affinity for the KRAS protein and effectively interact with its critical residues. The findings suggest that these compounds could act as potential inhibitors of the KRAS protein, opening up new avenues for cancer management.

## Figures and Tables

**Table 1:** List of top 10 screened natural compounds and their BE.

**Natural compounds**	**Binding energy (kcal/mol)**
ZINC32502206	-10.5
ZINC98363763	-10.1
ZINC85645815	-9.8
ZINC98364259	-9.7
ZINC95919082	-9.6
ZINC08918208	-9.6
ZINC06624383	-9.5
ZINC72325129	-9.4
ZINC19371265	-9.3
ZINC95909989	-9.3
AMG 510 (positive control)	-9.1

**Table 2:** Physicochemical and ADMET properties prediction of top 4 compounds.

**Molecule Property**	**Value**				**Unit**
	**ZINC98364259**	**ZINC32502206**	**ZINC85645815**	**ZINC98363763**	
Molecular Weight	355	475.16	328.09	356.01	kg/mol
Number of Heteroatoms	8	8	6	8	/
Number of Rotatable Bonds	6	3	4	5	/
Number of Rings	3	6	3	3	/
Number of HA	6	7	6	6	/
Number of HD	2	1	1	3	/
log KOW	0.21	4.41	3.19	-0.34	log-ratio
Absorption					
Caco-2 Permeability	-5.34	-5.46	-5.03	-5.22	log(cm/s)
HIA	70.06	74.35	74.67	71.22	%
Pgp Inhibition	37.51	49.76	44.39	38.94	%
log D7.4	1.37	2.01	2.19	1.53	log-ratio
Aqeuous Solubility	-4.08	-4.47	-4.76	-4.18	log(mol/L)
Oral Bioavailability	48.26	39.33	45.85	46.16	%
Distribution					Unit
BBB	35.28	22.36	28.65	32.25	%
PPBR	48.44	43.46	45.7	52.89	%
VDss	3.49	4.34	3.1	3.69	L/kg
Metabolism					
CYP2C9 Inhibition	33.96	63.34	71.02	28.46	%
CYP2D6 Inhibition	97.27	95.46	89.91	87.33	%
CYP3A4 Inhibition	36.25	26.98	40.26	36.17	%
CYP2C9 Substrate	31.82	36.79	35.81	32.4	%
CYP2D6 Substrate	46.02	59.07	65.68	50.4	%
CYP3A4 Substrate	46.05	42.4	36.74	42.29	%
Excretion					
Half-Life	64.64	145.94	80.52	60.9	hr
CL-Hepa	33.41	39.36	46.68	33.26	uL min-1 (106 cells)-1
CL-Micro	43.28	48.06	45.96	42.85	mL min-1 g-1
Toxicity					
hERG Blockers	38.05	45.18	39.9	37.4	%
Ames	52.01	49.73	48.37	54.37	%
DILI	44.26	38.01	44.81	43.89	%
LD50	2.31	2.77	2.32	2.23	-log(mol/kg)
